# Should I Take Aspirin? (SITA): randomised controlled trial of a decision aid for cancer chemoprevention

**DOI:** 10.3399/BJGP.2023.0385

**Published:** 2024-07-09

**Authors:** Shakira R Onwuka, Jennifer McIntosh, Finlay Macrae, Patty Chondros, Lucy Boyd, Rushani Wijesuriya, Sibel Saya, Napin Karnchanachari, Kitty Novy, Mark A Jenkins, Fiona M Walter, Lyndal Trevena, Javiera Martinez Gutierrez, Kate Broun, George Fishman, Julie Marker, Jon Emery

**Affiliations:** Centre for Cancer Research, University of Melbourne; Department of General Practice and Primary Care, University of Melbourne; Melbourne School of Population and Global Health, University of Melbourne, Melbourne, Australia.; Centre for Cancer Research, University of Melbourne; Department of General Practice and Primary Care, University of Melbourne; Melbourne School of Population and Global Health, University of Melbourne, Melbourne, Australia.; Colorectal Medicine and Genetics, Royal Melbourne Hospital, Australia; Department of Medicine, University of Melbourne, Melbourne, Australia.; Department of General Practice and Primary Care, University of Melbourne, Melbourne, Australia.; Centre for Cancer Research, University of Melbourne; Department of General Practice and Primary Care, University of Melbourne, Melbourne, Australia.; Clinical Epidemiology and Biostatistics Unit, Murdoch Children’s Research Institute, Australia; Department of Paediatrics, University of Melbourne, Melbourne, Australia.; Centre for Cancer Research, University of Melbourne; Department of General Practice and Primary Care, University of Melbourne, Melbourne, Australia.; Centre for Cancer Research, University of Melbourne; Department of General Practice and Primary Care, University of Melbourne, Melbourne, Australia.; Centre for Cancer Research, University of Melbourne; Department of General Practice and Primary Care, University of Melbourne, Melbourne, Australia.; National Health and Medical Research Council Professorial Fellow, cancer epidemiologist, Melbourne School of Population and Global Health, University of Melbourne, Melbourne, Australia.; Wolfson Institute of Population Health, Queen Mary University of London, London, UK; professor of primary care cancer research, Department of General Practice and Primary Care, University of Melbourne, Melbourne, Australia.; Primary Health Care, School of Public Health, University of Sydney, Sydney, Australia.; Centre for Cancer Research, University of Melbourne; Department of General Practice and Primary Care, University of Melbourne, Melbourne, Australia; School of Medicine, Family Medicine Department, Pontificia Universidad Católica de Chile, Santiago, Chile.; Prevention Division, Cancer Council Victoria, Melbourne, Australia.; PC4 Joint Community Advisory Group, University of Melbourne, Melbourne, Australia.; PC4 Joint Community Advisory Group, University of Melbourne, Melbourne, Australia.; Centre for Cancer Research, University of Melbourne; Department of General Practice and Primary Care, University of Melbourne, Melbourne, Australia; Wolfson Institute of Population Health, Queen Mary University of London, London, UK.

**Keywords:** aspirin, colorectal cancer, decision aid, general practice, informed decision making, randomised controlled trial

## Abstract

**Background:**

Australian guidelines recommend that people aged 50–70 years consider taking low-dose aspirin to reduce their risk of colorectal cancer (CRC).

**Aim:**

To determine the effect of a consultation with a researcher before an appointment in general practice using a decision aid presenting the benefits and harms of taking low-dose aspirin compared with a general CRC prevention brochure on patients’ informed decision making and low-dose aspirin use.

**Design and setting:**

Individually randomised controlled trial in six general practices in Victoria, Australia, from October 2020 to March 2021.

**Method:**

Participants were recruited from a consecutive sample of patients aged 50–70 years attending a GP. The intervention was a consultation using a decision aid to discuss taking aspirin to reduce CRC risk while control consultations discussed reducing CRC risk generally. Self-reported co-primary outcomes were the proportion of individuals making informed choices about taking aspirin at 1 month and on low-dose aspirin uptake at 6 months, respectively. The intervention effect was estimated using a generalised linear model and reported with Bonferroni-adjusted 95% confidence intervals (CIs) and *P*-values.

**Results:**

A total of 261 participants (86% of eligible patients) were randomised into trial arms (*n* = 129 intervention; *n* = 132 control). Of these participants, 17.7% (*n* = 20/113) in the intervention group and 7.6% (*n* = 9/118) in the control group reported making an informed choice about taking aspirin at 1 month, an estimated 9.1% (95% CI = 0.29 to 18.5) between-arm difference in proportions (odds ratio [OR] 2.47, 97.5% CI = 0.94 to 6.52, *P* = 0.074). The proportions of individuals who reported taking aspirin at 6 months were 10.2% (*n* = 12/118) of the intervention group versus 13.8% (*n* = 16/116) of the control group, an estimated between-arm difference of −4.0% (95% CI = −13.5 to 5.5; OR 0.68 [97.5% CI = 0.27 to 1.70, *P* = 0.692]).

**Conclusion:**

The decision aid improved informed decision making but this did not translate into long-term regular use of aspirin to reduce CRC risk. In future research, decision aids should be delivered alongside various implementation strategies.

## Introduction

In 2020, colorectal cancer (CRC) was the second most common cause of cancer deaths in Australia, and there were an estimated 1.9 million cases diagnosed globally.^1^^,^^2^ Meta-analyses of randomised controlled trials (RCTs) of low-dose aspirin have demonstrated reduced relative incidence and mortality of CRC by up to 25% and 33%, respectively.^3^ Meta-analyses of trials of aspirin for primary prevention of cardiovascular disease (CVD) demonstrate a reduced risk of ischaemic stroke, but an increased risk of non-fatal bleeding.^4^ The side effects of aspirin are well defined,^5^ but the likelihood of preventing death from cancer is 5–10 times greater than causing death from taking aspirin in this age group.^6^ These data informed Australian guidelines published in 2017, which recommend that clinicians consider prescribing low-dose aspirin for people aged between 50 and 70 years to prevent CRC.^7^

Decision aids are effective in general practice for communicating the benefits and risks of an intervention,^8^ particularly for preference-sensitive decisions. Decision aids can support informed choices about aspirin by individuals with Lynch syndrome (a genetic condition that increases cancer risk),^9^ and may also support this decision for the general population in primary care.

The Should I Take Aspirin? (SITA) trial is an efficacy trial of a consultation in general practice using a novel decision aid demonstrating the potential harms and benefits of low-dose aspirin for CRC and CVD prevention on informed decision making and low-dose aspirin uptake compared with general information about CRC.

## Method

Brief methods are presented here, which summarise the published trial protocol.^10^

**Table:** How this fits in

Australian guidelines recommend that people aged 50–70 years take low-dose aspirin to help prevent colorectal cancer (CRC). When the guidelines were published in 2017, there was no formal plan to implement them in clinical practice. This randomised controlled trial tested the use of a decision aid in general practice to communicate the benefits and harms of aspirin compared with general information about ways to prevent CRC. Additional implementation strategies with greater engagement with GPs may be necessary to increase aspirin use for CRC prevention.

### Study design and setting

This was a phase II, multi-site, parallel, two-arm individual RCT in six general practices in Victoria, Australia that was carried out from October 2020 to March 2021.

### Participant inclusion and exclusion criteria

Participants were eligible if they were aged 50–70 years, literate in written English, and provided informed consent. Exclusion criteria included taking low-dose aspirin or an anticoagulant regularly, a previous diagnosis of CRC, a known genetic predisposition to CRC, or an extensive family history suggesting a genetic predisposition.^11^ An extensive family history includes ≥2 first-or second-degree relatives on the same side with CRC, or ≥3 first-or second-degree relatives on the same side of the family with CRC or other Lynch syndrome-related cancer.^11^

### Recruitment

The practices provided researchers with appointment lists of patients who were in the eligible age range and had an appointment booked the following day to see if they might be interested in taking part in the SITA trial. Once contacted, if they were interested and eligible, the researcher invited them to attend a consultation either in a private consulting room at the general practice or using password-protected video-conferencing with them before their planned GP consultation. Informed consent was obtained during the consultation, followed by baseline data collection and randomisation, and depending on the trial arm, either the intervention or control protocol was delivered. Participants were informed that the trial was called ‘The Bowel Cancer Prevention Study’ and were not explicitly told that it was about aspirin. After this, patients had a consultation with their GP. Patients who refused or were ineligible were reassured that this would not be recorded in their medical records, and their clinical care would not be compromised.

### Intervention

The intervention involved a consultation delivered by a trained research assistant, in which the decision aid was discussed before the participant’s scheduled GP appointment. The decision aid is a sex-specific, tri-fold brochure that uses expected frequency trees to present the absolute changes in risk in people taking daily low-dose aspirin to the incidence of CRC, myocardial infarction, stroke, all-cause mortality, and gastrointestinal bleeding (see Supplementary Figures S1 and S2 for details).^10^ The decision aid refers to the Cancer Council Australia guidelines, prompts participants to discuss their decision with their GP before commencing low-dose aspirin, and lists contraindications for aspirin use.^12^ In response to the COVID-19 pandemic, an alternative teletrial model was developed that involved a video version of the decision aid,^10^ which was shown to all intervention participants.^13^

### Control

The control arm involved a consultation with participants before seeing their GP delivered by two researcher assistants, in which a ‘Reduce your bowel cancer risk’ tri-fold control brochure with an accompanying video was presented (see Supplementary Figure S3 for details). The control brochure and video focused on modifiable risk factors and CRC screening, with limited reference to low-dose aspirin.

### Changes to trial method

There was a deviation to the published protocol. In the protocol, a short message service (SMS) was planned to be sent 2 weeks after randomisation to remind intervention participants to discuss taking aspirin with their GP. However, at the end of the trial, it was discovered that the messages had not been automatically dispatched as a result of a technical issue and no one had received an SMS.

### Study outcomes and measures

Outcomes were measured at baseline before randomisation and at 1 and 6 months after randomisation. The 1 and 6 month follow-up questionnaires sent to participants can be found in the SITA trial protocol paper supplementary files I and J.^10^ The two co-primary outcomes were the difference between the study arms in the proportion of participants making an informed choice at 1 month and in the proportion who self-reported daily adherence to low-dose aspirin at 6 months.

#### Primary outcome: proportion of participants making an informed choice at 1 month

The multi-dimensional measure of informed choice (MMIC) was used to evaluate participants’ informed decision making regarding their self-reported behaviour of taking low-dose aspirin.^14^ An informed choice was considered to be one where the individual has sufficient knowledge about taking aspirin to prevent CRC and CVD, and where their behaviour (taking aspirin regularly in the past month or not) is concordant with their attitudes towards taking aspirin (positive or negative).^14^ Sufficient knowledge was defined using a total score (range 0 to 1), which was the sum of 11 statements about aspirin, for which the participant answered true, false, or unsure, plus one open-ended item (see Supplementary Box S1 for details). The threshold for sufficient knowledge was a score of 8.2 determined using the Angoff method.^15^ Attitude about taking aspirin consists of four items on a seven-point Likert scale.^14^ The total attitude scores range from 4 to 28, with higher scores reflecting a more negative attitude. The cut-off for a positive or negative attitude was set at the mid-point of the scale (positive attitude = 4–15; negative attitude = 16– 28).^14^

#### Primary outcome: proportion of participants who self-reported daily adherence to low-dose aspirin at 6 months

Participants were asked whether they had taken aspirin for at least 5 days per week, consistently, since consent (yes/no).

#### Secondary outcomes

Secondary outcomes included the differences between the study arms in:

1. mean decisional conflict scale at 1 month;^16^
2. the proportion of participants who self-reported daily adherence to low-dose aspirin at 1 month;
3. the proportion of participants who had discussed aspirin with their GP between baseline and 6 months, which was collected in an electronic medical record audit at 6 months by a research assistant blinded to participant allocation; and
4. the proportion of participants who reported behavioural changes made to reduce their risk of CRC at 1 and 6 months, including dietary changes, quitting smoking, screening for CRC, and whether they spoke to their GP about these changes.

#### Baseline measures

Participant demographics and CRC and CVD risk factors were collected at baseline. Family history was used to evaluate CRC risk considering close relatives diagnosed with CRC before age 55 years or multiple relatives diagnosed with CRC, indicating an elevated CRC risk, while self-reported risk factors including diabetes, high cholesterol, current use of high blood pressure medication, family history of CVD, and history of cigarette smoking indicated increased CVD risk for the participant.

Socioeconomic status was based on the Index of Relative Socio-economic Advantage and Disadvantage^17^ using the participant’s postcode of residence. The Subjective Numeracy Scale (SNS)^18^ was used to determine individuals’ preferences for numerical versus prose information. SNS is an eight-item questionnaire, where each item is rated on a Likert scale to calculate the total score, and then the average score is calculated. A higher score indicates a higher preference for numerical information. Other data collected were details of participants’ age, sex, country of birth, number of medications they were taking, education, and language spoken at home.

### Randomisation and blinding

Participants were randomly allocated 1:1 to the intervention or control arms using a computer-generated allocation sequence generated by the trial statistician and stratified by general practice, sex, and mode of intervention delivery (face-to-face or teletrial) using permuted blocks of random sizes of two and four within the stratum. GPs and research assistants who delivered the intervention and control could not be blinded but were not involved in the collection of follow-up data or data analysis. Before consenting, GPs were shown the decision aid and were made aware that their patients may ask about taking low-dose aspirin to prevent CRC. GPs were advised against changing their usual clinical practices during the trial.

Trial investigators were blinded to participant allocation. Participants were blinded and advised that they would be randomly assigned to one of two groups and, in either, they would receive information about reducing their CRC risk.

### Sample size

A total of 258 participants (129 per arm) were required to achieve 80% power with a two-sided Bonferroni-adjusted 2.5% alpha level for the two co-primary outcomes to estimate a minimum 20% between-arm difference in the proportion of participants regularly using low-dose aspirin at 6 months (39% versus 19%), and the proportion making an informed choice about low-dose aspirin use at 1 month (54% versus 34%). This allowed for 15% attrition at 6 months.

### Statistical analysis

The detailed statistical analysis plan (SAP) is available on the Australian New Zealand Clinical Trials Registry (ID: ACTRN12620001003965).^19^ All analyses were conducted using Stata (version 17).

Descriptive statistics were used to compare baseline participant demographic characteristics between the two study arms. Analysis was intention to treat for the two co-primary outcomes and secondary outcomes 1–3, where all randomised participants were included in the analysis using a multiple imputation approach (see Supplementary Box S2 for details). Exceptions were participants who explicitly withdrew their data before data analysis. For binary outcomes, logistic regression, adjusted for general practice (metropolitan versus rural), brochure type based on sex (male versus female), and mode of trial delivery (face-to-face or teletrial), was used to estimate the odds ratio (OR) (relative measure). Adjusted estimates of the between-arm difference in proportions (absolute measure) were generated using Stata margins command after fitting the logistic model.^20^ It was not possible to estimate the between-arm difference in proportions using the generalised linear model with the identity link function and binomial family as originally planned because of model convergence issues for several binary outcomes. The between-arm difference in means for the decisional conflict scale was estimated using linear regression adjusted for general practice, brochure type, and delivery mode. In addition, three sensitivity analyses were conducted:

1. adjusted for pre-specified baseline variables, general practice, sex, and mode of delivery using the same regression models;
2. the same as 1, adjusted for age in years and numeracy using the SNS; and
3. participants with complete follow-up only.

Estimates of the intervention effect were presented with Bonferroni-adjusted 95% confidence intervals (CIs) and *P*-values for two comparisons for the co-primary outcomes and with 95% CIs for all other secondary outcomes. The 97.5% CIs were used due to there being co-primary outcomes. The standard 95% CIs were used for the secondary outcomes and sensitivity analyses.

## Results

### Flow of participants in the trial and loss to follow-up

**Figure 1.**
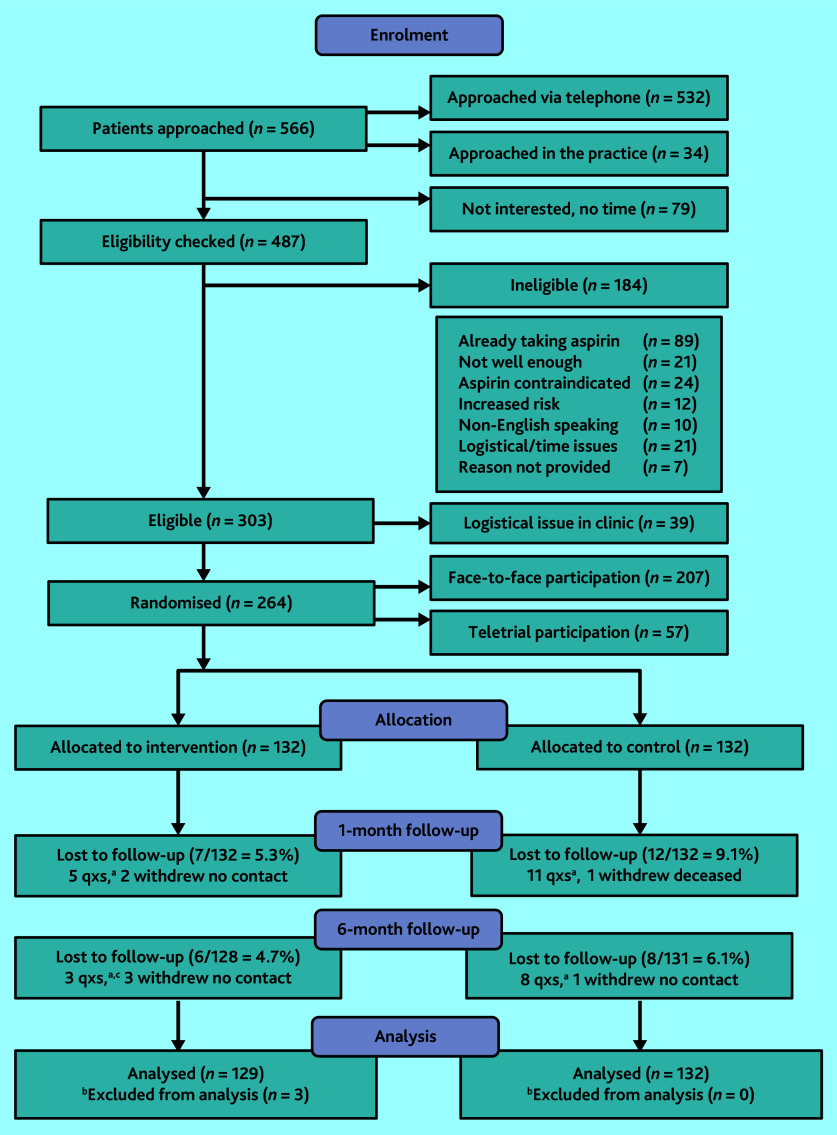
Participant flow diagram. ^a^Number of follow-up questionnaires not completed. ^b^Participants excluded after randomisation as researchers became aware of them taking blood thinners, which were contraindicated for aspirin, so they were excluded from the trial. ^c^ Follow-up questionnaires. qxs = questionnaires.

Between October 2020 and March 2021, 264 participants consented (87.1% of 303 eligible patients) from six general practices and were randomly allocated to the two trial arms (Figure 1). Three participants allocated to the intervention were found to be taking anticoagulants, which for the purposes of this trial were considered as contraindicated with taking low-dose aspirin and were excluded from analyses. Survey response rates were high at 85.6% and 89.2% at 1 and 6 months, respectively. Participant characteristics in the two arms were similar, apart from a family history of CVD or CRC (Table 1).

**Table 1.** Descriptive statistics of baseline characteristics for all participants and by study arm

**Variable**	**All participants, % (*n*)^a^**	**Intervention, % (*n*)^a^**	**Control, % (*n*)^a^**
**Total**	261	129	132

**Age, years, mean (SD)**	60.8 (6.3)	60.8 (6.3)	60.9 (6.3)

**Sex**			
Female	59.8 (156)	59.7 (77)	59.8 (79)
Male	39.8 (104)	39.5 (51)	40.2 (53)
Transgender	0.4 (1)	0.8 (1)	0 (0)

**Male or female decision aid**			
Female	59.8 (156)	59.7 (77)	59.8 (79)
Male	40.2 (105)	40.3 (52)	40.2 (53)

**IRSAD socioeconomic status**			
Disadvantaged 1	3.8 (10)	5.4 (7)	2.3 (3)
2	2.3 (6)	0.8 (1)	3.8 (5)
3	37.5 (98)	41.1 (53)	34.1 (45)
4	16.5 (43)	13.2 (17)	19.7 (26)
Advantaged 5	39.8 (104)	39.5 (51)	40.2 (53)

**Country of birth**			
Australia	67.8 (177)	67.4 (87)	68.2 (90)
Overseas	32.2 (84)	32.6 (42)	31.8 (42)

**Current medications, excluding vitamins**			
0	31.0 (81)	28.7 (37)	33.3 (44)
1	22.2 (58)	21.7 (28)	22.7 (30)
2–3	23.8 (62)	20.2 (26)	27.3 (36)
4–5	13.0 (34)	15.5 (20)	10.6 (14)
>5	10.0 (26)	14.0 (18)	6.1 (8)

**Education**			
Never completed high school	15.7 (41)	16.3 (21)	15.2 (20)
Completed high school only	18.8 (49)	20.9 (27)	16.7 (22)
TAFE qualifications or similar	21.1 (55)	17.1 (22)	25.0 (33)
University degree or higher	44.4 (116)	45.7 (59)	43.2 (57)

**Languages spoken at home**			
English	94.3 (246)	94.6 (122)	93.9 (124)
Other	5.7 (15)	5.4 (7)	6.1 (8)

**Subjective Numeracy Score, mean (SD)**	4.1 (1.2)	4.1 (1.3)	4.2 (1.1)

**Total**	261	129	132

**General practice**			
Metropolitan clinic 1	11.9 (31)	11.6 (15)	12.1 (16)
Regional clinic 2	4.2 (11)	4.7 (6)	3.8 (5)
Regional clinic 3	26.8 (70)	27.1 (35)	26.5 (35)
Metropolitan clinic 4	26.8 (70)	25.6 (33)	28.0 (37)
Metropolitan clinic 5	15.3 (40)	16.3 (21)	14.4 (19)
Metropolitan clinic 6	14.9 (39)	14.7 (19)	15.2 (20)

**Mode of trial delivery**			
Teletrial	21.1 (55)	20.9 (27)	21.2 (28)
Face-to-face	78.9 (206)	79.1 (102)	78.8 (104)

**Cardiovascular disease risks**			

**Family history of heart attack or stroke**			
Yes	55.9 (146)	51.9 (67)	59.8 (79)
No	38.7 (101)	41.9 (54)	35.6 (47)
Unsure	5.4 (14)	6.2 (8)	4.5 (6)

**Personal history of diabetes**			
Yes	7.3 (19)	8.5 (11)	6.1 (8)
No	92.3 (241)	90.7 (117)	93.9 (124)
Unsure	0.4 (1)	0.8 (1)	0 (0)

**Taking medication for high blood** **pressure**			
Yes	26.8 (70)	29.5 (38)	24.2 (32)
No	72.8 (190)	69.8 (90)	75.8 (100)
Unsure	0.4 (1)	0.8 (1)	0 (0)

**Personal history of high cholesterol**			
Yes	39.5 (103)	39.5 (51)	39.4 (52)
No	58.2 (152)	59.7 (77)	56.8 (75)
Unsure	2.3 (6)	0.8 (1)	3.8 (5)

**Current or history of smoking cigarettes**	47.5 (124)	47.3 (61)	47.7 (63)

**Family history of colorectal cancer^b^**			
Yes	3.8 (10)	1.6 (2)	6.1 (8)
No	92.7 (242)	95.3 (123)	90.2 (119)
Unsure	3.4 (9)	3.1 (4)	3.8 (5)

a *Unless otherwise stated.*

b *Participants were asked if they had more than one relative who had bowel cancer at any age, a family history of bowel cancer that did not meet the exclusion criteria for the trial. IRSAD = The Index of Relative Socio-economic Advantage and Disadvantage. SD = standard deviation.*

### Co-primary outcomes

Nearly 18% of participants in the intervention arm reported making an informed choice about taking low-dose aspirin compared with 7.6% in the control arm, an estimated increase of 9.1% (Bonferroni-adjusted 95% CI = 0.29 to 18.5; OR 2.47 [Bonferroni-adjusted 97.5% CI = 0.94 to 6.52; Bonferroni-adjusted *P* = 0.074]) (Table 2). There was no statistical evidence to support a difference in the proportion of participants reporting daily use of low-dose aspirin at 6 months between the intervention and control arms (10.2% versus 13.8%; between-arm difference of −4.0% [Bonferroni-adjusted 95% CI = −13.5 to 5.5]; OR 0.68 [Bonferroni-adjusted 97.5% CI = 0.27 to 1.70, Bonferroni-adjusted *P* = 0.346]). Similar results were observed in the sensitivity analyses; 41.6% of participants in the intervention groups had insufficient knowledge about taking aspirin, had a negative attitude about aspirin, and were not taking low-dose aspirin at 1 month (41.6% intervention; 60.2% control), forming the most common group of uninformed choices (Table 3).

**Table 2.** Co-primary outcomes and secondary outcomes by study arm for the SITA trial (*N* = 261)

**Co-primary outcomes**	**Intervention, *n* = 129, 49.4%, % (*n*/*N*)**	**Control, *n* = 132, 50.6%, % (*n*/*N*)**	**Estimated effect size**		

			**Difference (Bonferroni-adjusted 95% CI)**	**OR (Bonferroni-adjusted 97.5% CI)**	**Bonferroni-adjusted *P*-value**
Self-reported daily aspirin at 6 months	10.2 (12/118)	13.8 (16/116)			
Primary analysis^a^			–4.0 (–13.5 to 5.5)	0.68 (0.27 to 1.70)	0.692
Sensitivity analysis^b^			–3.1 (–12.4 to 6.3)	0.74 (0.29 to 1.88)	0.932
Sensitivity analysis^c^			–3.6 (–12.9 to 5.8)	0.70 (0.28 to 1.78)	0.790

Informed choice about taking aspirin at 1 month	17.7 (20/113)	7.6 (9/118)			
Primary analysis^a^			9.1 (0.29 to 18.5)	2.47 (0.94 to 6.52)	0.074
Sensitivity analysis^b^			9.6 (0.17 to 17.6)	2.70 (1.14 to 6.44)	0.048
Sensitivity analysis^c^			9.9 (0.31 to 19.5)	2.63 (1.00 to 6.93)	0.050

**Secondary outcomes**	**Intervention, % (*n*/*N*)^d^**	**Control, % (*n*/*N*)^d^**	**Difference (95% CI)**	**OR (95% CI)**	***P*-value**

Decisional conflict scale at 1 month, mean (SD) *n*	28.0 (16.4) 114	31.7 (18.8) 123			
Primary analysis^e^			–3.54 (–7.87 to 0.79)		0.109
Sensitivity analysis^f^			–3.73 (–8.08 to 0.62)		0.093
Sensitivity analysis^g^			–3.70 (–8.18 to 0.76)		0.104

Self-reported daily aspirin at 1 month	9.6 (11/115)	5.6 (7/125)			
Primary analysis^a^			3.7 (–3.6 to 11.0)	1.65 (0.61 to 4.45)	0.322
Sensitivity analysis^b^			3.9 (–2.8 to 10.6)	1.71 (0.63 to 4.60)	0.289
Sensitivity analysis^c^			4.1 (–2.6 to 10.7)	1.83 (0.68 to 4.95)	0.233

GP record audit, spoke to GP about taking aspirin at 6 months	17.5 (20/114)	9.0 (10/111)			
Primary analysis^a^			8.6 (–0.39 to 17.7)	2.09 (0.95 to 4.56)	0.066
Sensitivity analysis^b^			8.1 (–3.1 to 17.1)	2.60 (1.07 to 6.32)	0.035
Sensitivity analysis^c^			8.3 (–0.38 to 17.0)	2.13 (0.94 to 4.82)	0.069

a *Estimated using logistic regression adjusted for general practice, sex, and mode of delivery. Estimated using multiple imputation. Bonferroni-adjusted 95% confidence intervals and* P*-values reported for co-primary outcomes.*

b *Sensitivity analysis was the same as ‘^a^’, except also adjusted for age in years and numeracy scale. Estimated using multiple imputation.*

c *Sensitivity analysis was the same as ‘^a^’ using only participants that completed follow-up.*

d *Unless otherwise stated.*

e *Estimated using linear regression adjusted for general practice, sex, and mode of delivery. Estimated using multiple imputation.*

f *Sensitivity analysis was the same as ‘^e^’, except also adjusted for age and numeracy scale. Estimated using multiple imputation.*

g *Sensitivity analysis was same as ‘^e^’ using only participants who completed follow-up. Difference = difference in percentages between arms. OR = odds ratio. SD = standard deviation. SITA = Should I Take Aspirin?*

**Table 3.** Informed and uninformed choices across the three domains of the multi-dimensional measure for informed choice, at 1 month post-randomisation^a^

**Choices**	**Sufficient knowledge**	**Attitude**	**Behaviour**	**Intervention, *n* = 113, *n* (%)**	**Control, *n* = 118, *n* (%)**	**Total, *N* = 231, *n* (%)**
All possible informed choices						
1	✓	✓	✓	6 (5.3)	3 (2.5)	9 (3.9)
2	✓	✗	✗	14 (12.4)	6 (5.1)	20 (8.7)

All possible uninformed choices						
3	✓	✗	✓	0 (0)	0 (0)	0 (0)
4	✓	✓	✗	16 (14.2)	8 (6.8)	24 (10.4)
5	✗	✓	✓	3 (2.7)	3 (2.5)	6 (2.6)
6	✗	✗	✓	2 (1.8)	0 (0)	2 (0.9)
7	✗	✓	✗	25 (22.1)	27 (22.9)	52 (22.5)
8	✗	✗	✗	47 (41.6)	71 (60.2)	118 (51.1)

a *Tick marks (✓) indicate having sufficient knowledge, a positive attitude, and behaviour or a decision to take aspirin. Crosses (✗) indicate having insufficient knowledge, negative attitude, and behaviour or a decision to not take aspirin. Participants must have sufficient knowledge about aspirin for colorectal cancer prevention to make an informed choice. Additionally, they need to have an attitude concordant with their behaviour, that is, a positive attitude and a decision to take aspirin (choice 1), or a negative attitude, and a decision not to take aspirin (choice 2). All other combinations of knowledge, attitude, and behaviour are considered uninformed choices (choices 3 to 8).*

### Secondary outcomes

There was no statistical evidence to support between-arm differences in mean decisional conflict (Table 2). In the medical records audit, a higher proportion of intervention participants (17.5%) were identified as having discussed taking aspirin with their GP compared with 9.0% of controls (between-arm difference 8.6% [95% CI = −0.39 to 17.7]; OR 2.09 [95% CI = 0.95 to 4.56, *P* = 0.066]). Similarly, there was strong evidence that a greater proportion of participants in the intervention arm (30%) reported discussing aspirin with their GP than did control arm participants (12.0% and 15.1% of participants at 1 and 6 months, respectively) (Table 4). There was no statistical evidence to support between-arm differences in the proportion of participants for other self-reported behaviour change of other modifiable risk factors, which were included in the control brochure, except for self-reported discussion about screening for colorectal cancer at 1 month.

**Table 4.** Participant self-reported changed behaviours at 1 month and 6 months by study arm in the SITA trial

**Behaviours to reduce colorectal cancer risk**	**Intervention, *n* = 129, % (*n*/*N*)**	**Control, *n* = 132, % (*n*/*N*)**	**Estimated effect size^a^**		

			**Difference (95% CI)**	**OR (95% CI)**	***P*-value**
**Talked my GP about taking aspirin**					
1 month	30.4 (35/115)	12.0 (15/125)	18.4 (8.23 to 28.41)	3.45 (1.69 to 7.05)	<0.001
6 months	30.5 (36/118)	15.1 (18/119)	15.4 (5.21 to 26.14)	2.52 (1.31 to 4.85)	0.005

**Changed my diet**					
1 month	28.7 (33/115)	24.8 (31/125)	3.9 (–7.74 to 14.52)	1.19 (0.66 to 2.14)	0.563
6 months	33.9 (40/118)	35.0 (42/120)	–1.1 (–13.56 to 10.30)	0.94 (0.54 to 1.63)	0.814

**Talked to my GP about quitting smoking**					
1 month	18.5 (10/54)	14.0 (8/57)	4.5 (–7.20 to 19.94)	1.34 (0.42 to 4.31)	0.619
6 months	11.3 (6/53)	17.0 (9/53)	–5.7 (–17.26 to 8.34)	0.50 (0.14 to 1.82)	0.292

**Quit smoking^b^**					
1 month	30.9 (17/55)	27.6 (16/58)	3.3 (–1.30 to 20.27)	1.11 (0.47 to 2.62)	0.802
6 months	42.6 (23/54)	52.8 (28/53)	–10.2 (–31.19 to 5.93)	0.67 (0.30 to 1.50)	0.331

**Spoke to GP about screening for colorectal cancer by FOBT**					
1 month	29.6 (34/115)	17.1 (21/123)	12.5 (2.73 to 23.57)	2.17 (1.16 to 4.04)	0.015
6 months	22.9 (27/118)	26.7 (32/120)	–3.8 (–14.18 to 7.78)	0.83 (0.45 to 1.51)	0.537

**Completed FOBT test**					
1 month	13.9 (16/115)	14.4 (18/125)	–0.5 (–0.93 to 0.83)	0.95 (0.45 to 1.99)	0.895
6 months	23.7 (28/118)	28.6 (34/119)	–4.9 (–15.78 to 6.34)	0.77 (0.42 to 1.39)	0.380

**Spoke to GP about screening for colorectal cancer by colonoscopy**					
1 month	14.8 (17/115)	14.5 (18/124)	0.3 (–10.33 to 9.37)	0.97 (0.46 to 2.01)	0.925
6 months	20.5 (24/117)	20.8 (25/120)	–0.4 (–10.48 to 10.05)	1.03 (0.53 to 1.98)	0.940

**Had a colonoscopy**					
1 month	11.2 (13/116)	6.5 (8/124)	4.7 (–2.44 to 11.63)	1.79 (0.70 to 4.55)	0.225
6 months	13.6 (16/118)	11.7 (14/120)	1.9 (–6.31 to 10.32)	1.22 (0.57 to 2.63)	0.611

a *Estimated using logistic regression for each outcome, adjusted for general practice, sex, and mode of delivery using only participants who completed follow-up.*

b *This question was only asked to people who either had a history of smoking cigarettes or smoked cigarettes at baseline. There were 63 participants in the intervention arm and 61 in the control arm.* n *= number of participants who self-reported ‘yes’ to each of the behaviours.* N *= total number of participants who provided a response to this item in the follow-up questionnaires. FOBT = faecal occult blood test. SITA = Should I Take Aspirin?*

## Discussion

### Summary

To the authors’ knowledge, this is the first trial to assess the efficacy of a decision aid to support discussions about low-dose aspirin to prevent CRC in an average-risk general practice population. There is a long history of aspirin being recommended to prevent CVD and stroke, although most international guidelines now recommend this only for secondary prevention.^21^^–^24 Meta-analyses of aspirin trials for CVD prevention and CRC informed the Australian CRC chemoprevention guidelines,^7^ but implementation plans were lacking. The researchers developed the first sex-specific decision aids for low-dose aspirin use as a potential route for clinical implementation of these guidelines.^25^

This RCT showed an increase in participants’ mean knowledge scores and informed decisions about taking low-dose aspirin to prevent CRC at 1 month, with a higher proportion of participants discussing taking aspirin with their GP in the intervention arm, but with little impact on uptake of low-dose aspirin after 6 months. Most participants who made informed choices decided not to take low-dose aspirin, and the estimated between-arm difference of 9.1% in the proportion making an informed choice to take low-dose aspirin at 1 month fell below the predetermined minimum threshold of 20%, which was considered clinically important by trial investigators.

### Strengths and limitations

Individuals were randomised as the risk of contamination was expected to be low based on a similar trial,^26^ and the intervention was delivered at an individual level, further reducing the risk of contamination because it targeted individuals rather than groups or communities, making it less likely for control group members to be inadvertently exposed to the intervention. Further, a larger sample size would have been required if the unit of randomisation was the practice. To minimise the risk of contamination in the control arm, the trial’s focus on aspirin was concealed from all participants. However, the intervention effect may have been attenuated through multiple questionnaires about aspirin use to participants. GPs’ involvement in the trial might have raised their awareness of aspirin guidelines and led them to discuss this with their patients. Overall, 15% of control participants reported a discussion about aspirin with their GP and, even though more participants in the intervention arm (30%) reported discussing taking low-dose aspirin with their GP, the content of those discussions is not known, neither is how GPs’ attitudes towards aspirin might have influenced patients’ decisions. Fewer discussions about aspirin were recorded in participants’ medical records than were self-reported by participants. This might be a result of social desirability bias influencing participants’ self-reported responses, or GPs not referring to this conversation in their records.

Recruitment and retention rates of trial participants were high, achieved with the use of a novel teletrial and an adapted protocol for online trial delivery during the COVID-19 pandemic.^13^ A limitation of the efficacy trial design was that it was not possible to determine whether the use of the decision aid by a practice nurse or GP, rather than in a standardised way by a researcher, would result in increased low-dose aspirin use.

Most participants did not demonstrate sufficient knowledge to make an informed decision based on the MMIC measure. A limitation of the MMIC is the need to define a binary cut-point on the knowledge and attitude scales. The Angoff method was used, in which a group of researchers and clinicians reached a consensus on the cut-point for sufficient knowledge. The cut-point for sufficient knowledge may have been set too high (higher than the midpoint for knowledge score), resulting in a lower proportion of participants classified as making informed choices. The MMIC was measured at 1 month to allow sufficient time to observe a behaviour change (taking aspirin). Intervention participants might have potentially reduced knowledge scores at 1 month than if surveyed immediately on receipt of the intervention, which may have attenuated the estimated intervention effect. Retained knowledge may be a more important measure of informed decision making than short-term recall.

### Comparison with existing literature

To date, only one decision aid about using aspirin for primary prevention of CRC has been evaluated and shown to be acceptable to GPs and pharmacists, but its effectiveness in improving low-dose aspirin uptake and patient-informed choice has not been tested in a trial.^27^ A systematic review of decision aids for complex healthcare decisions found that they increased knowledge, facilitated discussions between clinicians and patients, and reduced decisional conflict, but their effect on informed decision making was less consistent across trials.^8^ In the current RCT, the proportion of intervention participants reporting making an informed choice about taking aspirin increased by 9.1% compared with control participants at 1 month, but the overall proportion making informed choices was low. Most participants had insufficient knowledge, leading them not to make informed choices about taking aspirin.

### Implications for research and practice

This RCT of a decision aid to implement aspirin guidelines to prevent CRC led to differences in participants’ knowledge and informed choice, and prompted discussions between patients and GPs; however, there was no difference in aspirin uptake between the study arms. Since the Australian guidelines recommending low-dose aspirin for CRC prevention were published in 2017,^7^ the ASPREE trial results cast doubt on the benefits of low-dose aspirin in primary prevention for many conditions in healthy Australians aged >70 years.^28^ Furthermore, the US Preventive Services Task Force has modified its recommendations about the use of aspirin for CRC prevention due to including aspirin trials with short-term follow-up into their systematic review of the evidence.^29^ Although the ASPREE trial involved an older population than the Australian guidelines that recommend considering aspirin, the publicity and media coverage surrounding the ASPREE trial in Australia possibly created confusion for GPs and the general public at the time of this trial. The decision aid described in this RCT was designed to clarify the evidence about the relative benefits and harms of low-dose aspirin for primary prevention of CRC in people aged 50–70 years for both patients and GPs. To implement the guidelines, other interventions in addition to decision aids may need to be tested. This may require GPs to be more confident in the strength of evidence underpinning the aspirin recommendations before they are comfortable supporting patients in taking regular aspirin to prevent CRC and other long-term conditions.

## Data

Data are available on request from the corresponding author.
